# Early identification of emergency abdominal surgery candidates in the emergency department – a Delphi study

**DOI:** 10.1007/s00423-026-04058-7

**Published:** 2026-04-24

**Authors:** Dorte Melgaard, Jakob F. Jørgensen, Thomas Stilling-Vinther, Anne Lund Krarup, Mathias Håkon Madsen, Luit Penninga

**Affiliations:** 1 Department of Emergency Medicine and Trauma Centre, EMRUn, Aalborg University Hospital, Hobrovej 18-22, Aalborg, Denmark; 2 Department of Clinical Medicine, Aalborg University, Aalborg, Denmark; 3 Department of Gastrointestinal Surgery, Aalborg University Hospital, Aalborg, Denmark

**Keywords:** Bowel ischemia, Perforation, Peritonitis, Indicators, AHA

## Abstract

**Purpose:**

Acute high-risk abdominal (AHA) surgery is associated with considerable morbidity and mortality. In the emergency department (ED), early identification of patients requiring AHA surgery remains challenging and is often based on non-standardized clinical judgment. This study aimed to identify clinical indicators that may support the timely recognition of patients in need of emergency abdominal surgery.

**Methods:**

A modified Delphi study was conducted to achieve expert consensus on early clinical indicators of AHA surgery. Two Delphi rounds were performed. In round one, experienced surgeons and emergency clinicians rated 30 predefined indicators as insignificant, less important, important, or very important. Consensus was defined as ≥ 75% of participants rating an indicator as important or very important. In round two, participants were asked to rank the importance of the indicators rated as “important” or “very important” in round one.

**Results:**

A total of 87 surgeons and emergency physicians participated in round one, with 62 (71%) completing round two. Consensus was reached for 11 indicators considered important for identifying AHA patients on arrival in the ED, e.g. acute pain onset within minutes, VAS pain score 6–10, repeated vomiting, and absence of bowel movement within 24 h. In round two, clinicians ranked these indicators; however, no clear consensus emerged regarding their relative prioritisation.

**Conclusion:**

This Delphi study identified 11 clinically relevant indicators for the early identification of patients requiring AHA surgery in the ED. The study underscores the complexity of early recognition and supports the need for structured, data-informed screening tools to aid clinical decision-making.

## Introduction

Acute high-risk abdominal (AHA) surgery encompasses a heterogeneous group of emergency procedures performed for life-threatening intra-abdominal conditions, including bowel ischemia, perforation, and peritonitis. The 30-day mortality rates for these conditions range from 15% to over 30% in some cohorts [1, 2]. Timely surgical intervention is critical in managing patients requiring AHA surgery. Delays in recognition and referral, particularly from the emergency department (ED), have been consistently associated with worse outcomes, such as increased rates of septic shock, multiorgan failure, and death [3, 4]. However, early identification of patients who may require emergency abdominal surgery remains a significant challenge. While established screening tools such as the National Early Warning Score (NEWS) and the Sequential Organ Failure Assessment (SOFA) score are designed to detect clinical deterioration in hospitalized patients, no dedicated tool currently exists for the early identification of patients with acute high-risk abdominal (AHA) conditions. The Delphi-derived indicators proposed in this study aim to address this gap, particularly in the early phase of ED decision-making, before such scoring systems become applicable [5, 6]. Current triage and assessment processes in the ED often rely on non-standardized clinical judgment, which can vary widely between providers and institutions [7]. Additionally, the often subtle and nonspecific presentation of abdominal emergencies in elderly and frail populations complicates timely diagnosis and decision-making [8]. These limitations highlight the need for a more structured approach to the early identification of patients on an AHA trajectory. A critical gap remains in the preoperative phase: specifically, the early recognition and prioritization of clinical observations that suggest the need for urgent surgical consultation. Defining such clinical indicators could reduce time to diagnosis, facilitate earlier intervention, and shorten the time to surgery. Early surgery reduces both mortality and morbidity, improving health-related quality of life and reducing costs to the healthcare system.

This study aimed to identify and prioritize early clinical indicators for AHA surgery through a Delphi consensus process involving abdominal surgeons and emergency physicians.

## Materials and methods

### Study Design

This study employed a modified Delphi technique to achieve expert consensus on early clinical indicators for the need for AHA surgery. The Delphi method is a structured, iterative process that uses questionnaires and controlled feedback, conducted in accordance with best-practice guidelines for Delphi studies in healthcare [9–11]. We utilized the Delphi Critical Appraisal Tool to enhance the quality of the study, adhering to the 16-item Checklist [12]. The study was initiated by authors who are experienced doctors in abdominal surgery and emergency medicine, as well as researchers in the field.

### The two survey rounds

#### Round 1

For the first round, a working group (*n* = 7) of abdominal surgeons and emergency physicians developed an initial list of 30 candidate early clinical indicators (Table 1). These were informed by a scoping review of the literature, including studies on high-risk surgical pathways (Tengberg, Bay-Nielsen, et al., 2017; Timan et al., 2020), national and international guidelines on emergency abdominal surgery, a non-validated screening tool used in the Region of Southern Denmark, and the working group’s clinical experience.

**Table 1:** List of the 30 indicators for early identification of patients who may require AHA surgery

Pain onset – hyperacute within seconds	No bowel movement or passage of flatus for < 12 h	Clinical Frailty Scale > 6
Pain onset – acute within minutes	No bowel movement or passage of flatus for < 24 h	Age > 65 years
Pain duration < 12 h	Absence of bowel sounds (auscultated for at least 2 min)	Age > 75 years
Pain duration < 24 h	Pale	Comorbidity – more than two conditions
Pain duration > 3 days	Cold, clammy sweating	Sudden functional decline within 24 h
Diffuse pain	Fever	Worsening abdominal pain with sudden movement
Migrating pain	Tachycardia	Increasing abdominal girth within 12 h
VAS 6–10	Systolic blood pressure < 100 mmHg	Known history of previous ileus or peptic ulcer disease
Nausea	Previous surgery for AHA	Hospital admission within the past 7 days
Recurrent vomiting	Previous abdominal surgery – not AHA	GCS < 15

Indicators included physiological parameters and clinical signs that are easily and potentially observable in the emergency department. The indicators were sent as an electronic questionnaire. Participants received the survey and were asked to assess the relevance of each item for the early identification of patients who may require AHA surgery. All 30 indicators were rated on a 4-point Likert scale (0 = not important, 1 = somewhat important, 2 = important, 3 = very important). As commonly accepted in the literature, an indicator was considered to have reached consensus for inclusion if ≥ 75% of respondents rated it as “important” or “very important” (scores of 2 or 3) [13]. It was also possible for participants to note if they believed there was an indicator that should be included.

Eligible participants were recruited from the existing professional networks of the study authors and met the inclusion criteria by having a background in emergency medicine or abdominal surgery, either during or after specialty training including members of academic societies, conference attendees, and professional collaborators. To ensure a broad representation of clinical expertise, panelists were selected from several academic and regional hospitals across all five Danish regions to ensure representativeness and diversity of clinical perspectives. While efforts were made to recruit participants with varied backgrounds and experiences, the reliance on the authors’ professional networks may have introduced a degree of selection bias, as individuals within these networks may not represent the full spectrum of expertise in emergency abdominal surgery or emergency medicine. Moreover, panelists from certain regions or hospitals may have different experiences and practices that could influence their perspectives. Additionally, although we aimed for a diverse selection of experts, those with more prominent or established careers may have been overrepresented, potentially skewing the consensus toward the views of more experienced professionals.

The first-round survey was distributed by email on 28 August 2025, and recipients were encouraged to disseminate it through relevant channels, networks, forums, workplaces, and to colleagues who met the inclusion criteria. Participation was voluntary, and anonymity was maintained throughout the process to minimize potential bias or influence from dominant voices [14]. The Delphi process was conducted using the online survey platform Research Electronic Data Capture system (REDCap) [15, 16]. Prior to distribution, the survey was pilot-tested for functionality by members of the working group.

#### Round 2

The working group reviewed the findings from round one and planned round two of the survey. In this round, participants were asked to rank the indicators that had achieved more than 75% consensus based on their importance in identifying a patient as requiring AHA surgery [17]. Drawing on qualitative comments from round one and the clinical experience of the working group, an additional indicator was added: “Does the nurse in the Emergency Department assess that the patient is at risk of requiring surgery?” This brought the total number of indicators to 11. The revised survey was pilot-tested for functionality by the working group before being emailed on 24 October 2025 to all participants who had responded in round one. A reminder was sent on 18 November 2025. In the second round, participants were asked to rank the importance of each indicator on a scale, with each numerical rank used only once.

### Data Analysis

Quantitative data were analyzed using descriptive statistics. The level of agreement for each indicator was calculated as the proportion of respondents assigning a score of 2 or 3. Qualitative comments were reviewed and thematically analyzed to provide context and inform interpretation. The final list of indicators consisted of those that met the predefined consensus threshold by the end of the Delphi rounds. Data from the second round were presented as median scores with interquartile ranges for each indicator.

### Ethical Considerations

This study involved consultation with expert clinicians and did not include patient data. Therefore, it was deemed exempt from full ethical review under institutional guidelines. Informed consent was obtained electronically from all participants, and participation was anonymous and voluntary in accordance with ethical principles for Delphi studies.

## Results

A total of 87 participants responded in round one, and 62 (71%) participated in round two, with an almost equal distribution between the two specialties, as shown in Table 2.

**Table 2:** Demography of the participants

	Round 1 *N* = 87	Round 2 *N* = 62
Medical specialty:		
Emergency medicine	41 (47%)	30 (53%)
Surgery	46 (53%)	27 (47%)
Position:		
Junior doctor	34 (39%)	20 (35%)
Consultant	11 (13%)	9 (15%)
Senior consultant	36 (42%)	24 (42%)
Professor and senior consultant	3 (3%)	3 (5%)
Other	3 (3%)	2 (3%)
Regions:		
Capital Region	11 (13%)	6 (10%)
Zealand	12 (14%)	7 (12%)
Southern Denmark	19 (22%)	15 (26%)
Central Denmark	22 (25%)	16 (28%)
Northern Denmark	23 (26%)	14 (24%)

Round 2 missing information from 5 participants

**Fig. 1:**
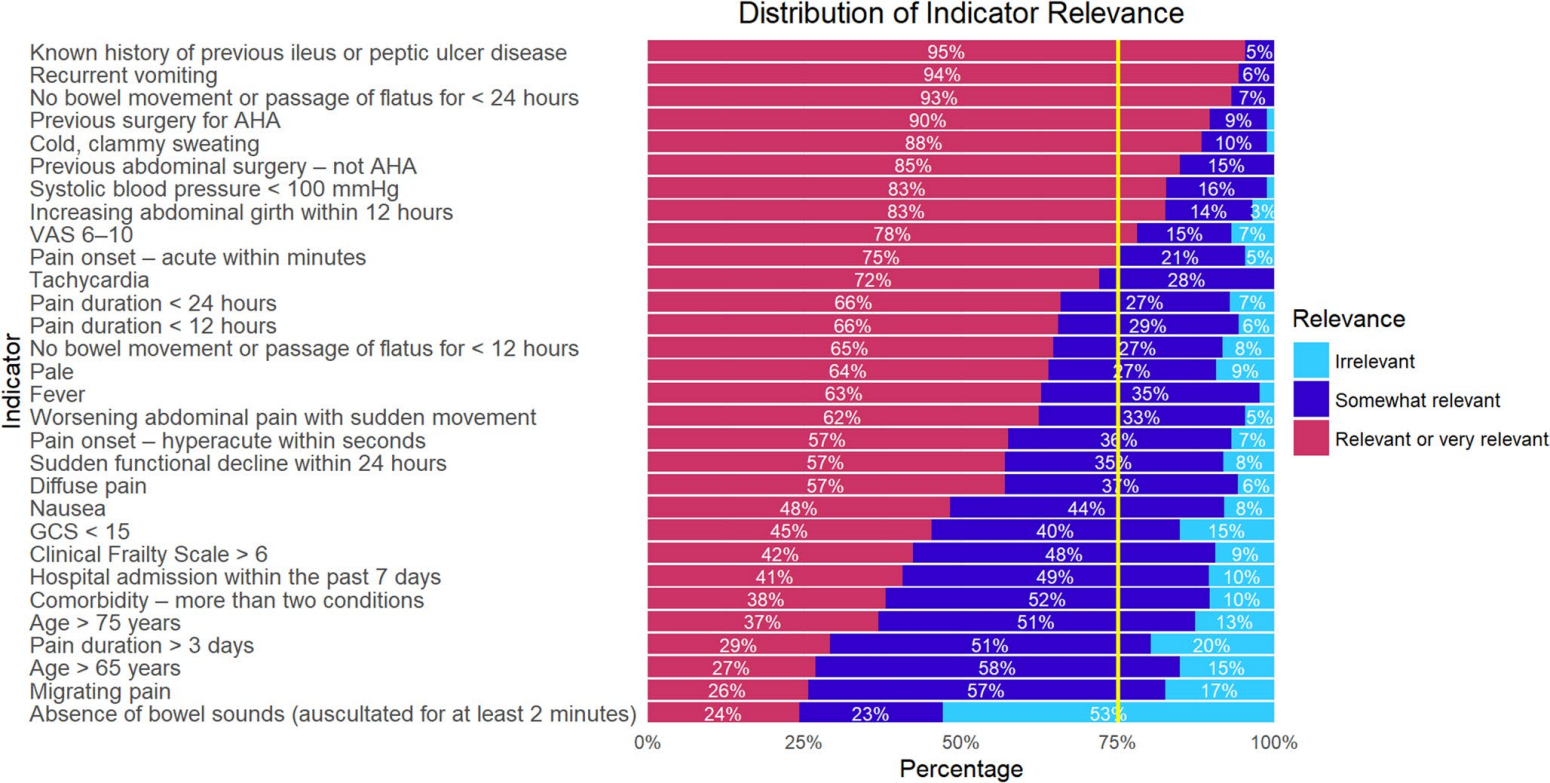
Clinician consensus on early indicators important for identifying AHA in the ED. Consensus of importance was depicted as a yellow line (75% or more)

As shown in Fig. 1, consensus was reached on the following 10 indicators being important or very important for identifying AHA patients upon arrival in the ED: known history of previous AHA condition treated conservatively, recurrent vomiting, no bowel movement or passage of flatus < 24 h, previous surgery for AHA, cold and clammy sweating, previous abdominal surgery (not AHA), systolic blood pressure < 100 mmHg, increased abdominal girth within 12 h, VAS 6–10, and acute pain onset within minutes.

**Fig. 2:**
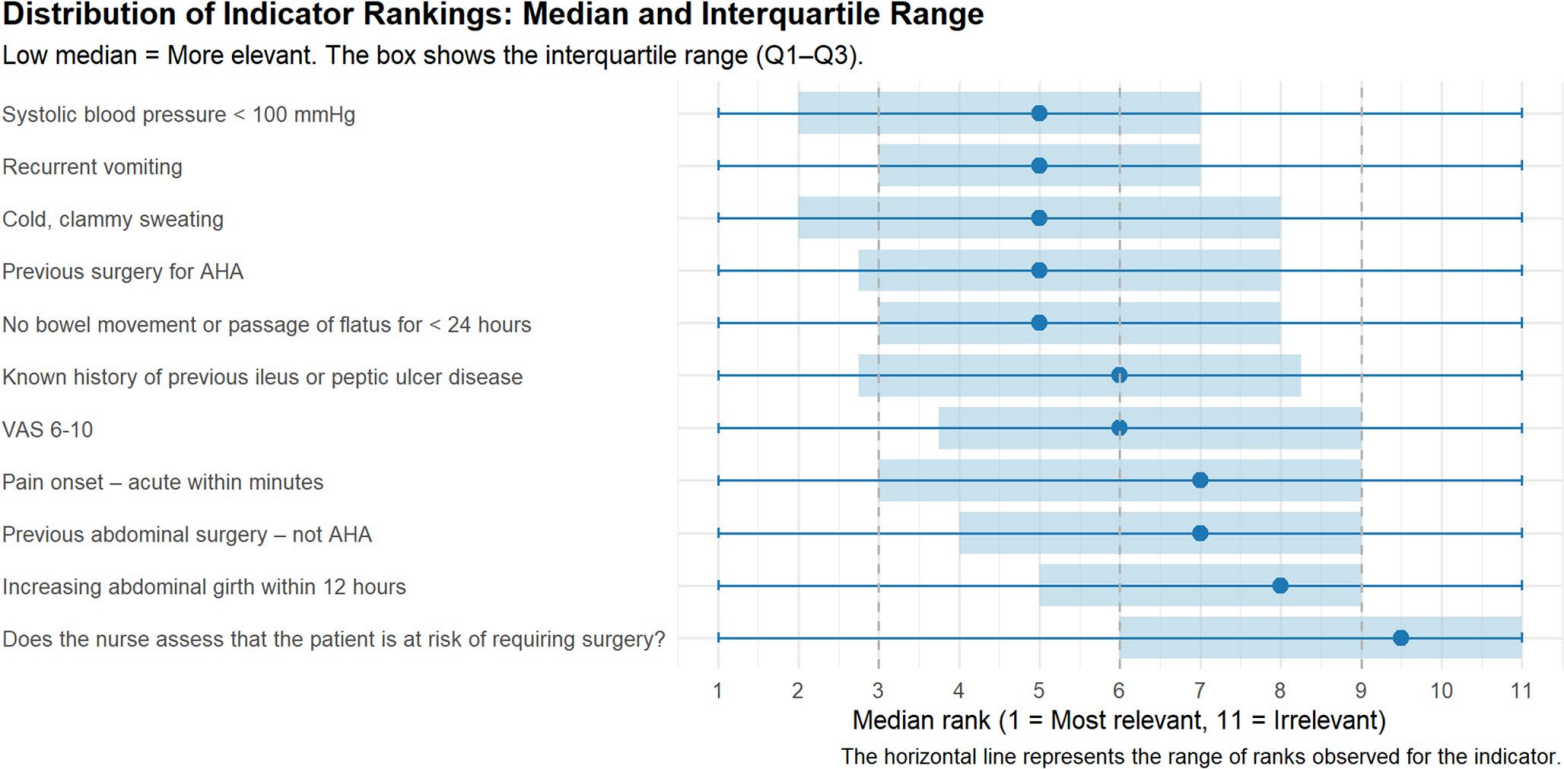
Distribution of indicator rankings

In the second round, clinicians were asked to rank the ten indicators that had reached consensus in round one, along with the additional indicator, “Does the nurse assess that the patient is at risk of requiring surgery?“, based on their importance for the early identification of AHA. No clear agreement emerged regarding which indicators were considered more important, as illustrated in Fig. 2.

## Discussion

We conducted a two-round Delphi Consensus study to identify emergency abdominal surgery candidates in the Emergency Department. In Round 1, consensus was reached for 11 indicators that identified high-risk patients. In the second round, no clear consensus emerged regarding the relative importance of the indicators, as they were ranked at comparable levels.

In Denmark, a national collaborative working group has been working over the last ten years to improve outcomes for acute high-risk abdominal surgical patients [18]. The project includes a prospective nationwide registry of all acute high-risk abdominal surgical patients in Denmark Early and accurate identification of patients with conditions requiring acute high-risk abdominal surgery is considered of utmost importance [19]. How to properly develop a screening tool to identify these patients has been discussed, but considered to be very difficult [18]. Consequently, proper identification continues to rely on clinical judgment by an experienced physician or surgeon, leading to delays, as not all patients are assessed by an experienced clinician upon arrival in the ED. This raised the question of whether it is possible to identify reliable indicators for patients with conditions requiring emergency abdominal surgery, as these conditions are heterogeneous (e.g., ischemia, perforation, bowel obstruction) and differ in clinical presentation and symptoms. Even patients with the same condition may present in different ways, with varying symptoms. Few studies have assessed the factors and clinical indicators that predict the need for in-hospital emergency interventions as an outcome [20]. These studies primarily focus on trauma patients, where predictors for in-hospital emergency interventions and surgery were identified, leading to the development of triage models [21–23]. However, we have not identified similar ‘predictive model’ studies for emergency non-trauma abdominal surgery.

In this study, consensus was reached that ten clinical indicators are considered important or very important for identifying patients requiring acute high-risk abdominal (AHA) surgery upon arrival in the emergency department: rapid onset of acute pain, high pain intensity (VAS 6–10), repeated vomiting, absence of bowel movement within 24 h, clammy sweating, systolic blood pressure below 100 mmHg, previous AHA surgery, prior abdominal surgery unrelated to AHA, known AHA treated conservatively, and increased abdominal girth. These findings are consistent with earlier studies that highlight the challenges of early recognition of patients at risk for severe abdominal pathologies such as bowel ischemia, perforation, and peritonitis [24, 25]. For instance, hypotension and tachycardia have been repeatedly associated with poor outcomes in AHA patients, underscoring the relevance of systolic blood pressure as an early warning sign [26]. Similarly, the acute onset of severe pain and gastrointestinal symptoms, such as vomiting and bowel obstruction, have been consistently reported as key red flags for conditions requiring urgent surgical intervention [27, 28]. Indicators reflecting surgical history, including prior AHA surgery or previous abdominal procedures, are also supported by existing literature, which highlights the increased risk of complications or recurrent pathology in these patients. Finally, signs of systemic compromise, such as clammy sweating and increased abdominal girth, align with previous observations that physiological derangements often precede overt clinical deterioration [28]. Collectively, these indicators offer a structured framework for early identification, supporting the development of standardized assessment tools in the emergency setting and pinpointing areas where clinical vigilance can be focused to improve outcomes for high-risk abdominal patients.

The clinical challenge is that these symptoms occur far more frequently in patients who do not require high-risk surgery (e.g., those with pyelonephritis). To our knowledge, no evidence-based tools for the early identification of patients with acute high-risk abdominal conditions (AHA) have been developed. This study is the first to convene a large panel of experienced clinicians in surgery and emergency medicine to determine which indicators they expect to be most useful for identifying patients requiring urgent surgical intervention.

The inability to rank the 11 identified indicators likely reflects the fact that such patients often present with subtle and nonspecific symptoms. Furthermore, it appears that no single indicator is decisive; rather, it is the combined pattern of multiple symptoms and objective clinical markers that facilitates identification. Beforehand, we already had serious doubts that experts would be able to rank the indicators in a similar way, and this was confirmed by our findings.

The next step will be to develop a screening tool based on these indicators. According to the findings from the second round of this study, the 11 indicators identified in the first round should be given equal weight in the screening tool. The results of the Delphi study will be combined with a review of patient records from electronic health records to identify which indicators are most commonly associated with patients requiring AHA surgery, as compared to those with abdominal pain who do not need emergency surgery. Together, the findings from the Delphi study and the patient record review will form the basis for the new screening tool.

Strengths and limitations.

The strength of our study lies in the fact that we designed and conducted it in accordance with the Delphi Consensus Assessment tool and adhered to the 16-item checklist, including the core DACT items: Anonymity, Iteration, Statistical Summary, and Controlled Feedback. A total of 87 and 62 experienced physicians participated in the respective rounds, representing a large panel from all regions of Denmark, which further strengthens the validity of our findings [29]. A limitation of the study is the small sample size, which restricts the analysis of differences between surgeons and emergency physicians, as well as variations by experience level, from being fully meaningful.

## Conclusion

This Delphi study identified 11 clinical indicators that experienced abdominal surgeons and emergency medicine physicians consider relevant for the early identification of patients requiring surgery for acute high-risk abdominal conditions (AHA). However, no consensus emerged on their relative importance, suggesting that early recognition depends on patterns of multiple symptoms and objective clinical markers, rather than any single factor. Combined with data review, these findings provide a foundation for the development, evaluation, and validation of a screening tool to support earlier identification of patients in need of AHA surgery.

## Data Availability

Data are available upon request from the corresponding author.
